# Bone marrow lesions from osteoarthritis knees are characterized by sclerotic bone that is less well mineralized

**DOI:** 10.1186/ar2601

**Published:** 2009-01-26

**Authors:** David J Hunter, Lou Gerstenfeld, Gavin Bishop, A David Davis, Zach D Mason, Tom A Einhorn, Rose A Maciewicz, Pete Newham, Martyn Foster, Sonya Jackson, Elise F Morgan

**Affiliations:** 1 Division of Research, New England Baptist Hospital, 125 Parker Hill Avenue, Boston, MA 02120, USA; 2 Orthopedic Department, Boston University, 715 Albany Street, Boston, MA 02118, USA; 3 Respiratory & Inflammation AstraZeneca, Macclesfield, Cheshire SK10 4TF, UK; 4 AstraZeneca R&D, Charnwood, Loughborough, Leicestershire LE11 5RH, UK

## Abstract

**Introduction:**

Although the presence of bone marrow lesions (BMLs) on magnetic resonance images is strongly associated with osteoarthritis progression and pain, the underlying pathology is not well established. The aim of the present study was to evaluate the architecture of subchondral bone in regions with and without BMLs from the same individual using bone histomorphometry.

**Methods:**

Postmenopausal female subjects (n = 6, age 48 to 90 years) with predominantly medial compartment osteoarthritis and on a waiting list for total knee replacement were recruited. To identify the location of the BMLs, subjects had a magnetic resonance imaging scan performed on their study knee prior to total knee replacement using a GE 1.5 T scanner with a dedicated extremity coil. An axial map of the tibial plateau was made, delineating the precise location of the BML. After surgical removal of the tibial plateau, the BML was localized using the axial map from the magnetic resonance image and the lesion excised along with a comparably sized bone specimen adjacent to the BML and from the contralateral compartment without a BML. Cores were imaged via microcomputed tomography, and the bone volume fraction and tissue mineral density were calculated for each core. In addition, the thickness of the subchondral plate was measured, and the following quantitative metrics of trabecular structure were calculated for the subchondral trabecular bone in each core: trabecular number, thickness, and spacing, structure model index, connectivity density, and degree of anisotropy. We computed the mean and standard deviation for each parameter, and the unaffected bone from the medial tibial plateau and the bone from the lateral tibial plateau were compared with the affected BML region in the medial tibial plateau.

**Results:**

Cores from the lesion area displayed increased bone volume fraction but reduced tissue mineral density. The samples from the subchondral trabecular lesion area exhibited increased trabecular thickness and were also markedly more plate-like than the bone in the other three locations, as evidenced by the lower value of the structural model index. Other differences in structure that were noted were increased trabecular spacing and a trend towards decreased trabecular number in the cores from the medial location as compared with the contralateral location.

**Conclusions:**

Our preliminary data localize specific changes in bone mineralization, remodeling and defects within BMLs features that are adjacent to the subchondral plate. These BMLs appear to be sclerotic compared with unaffected regions from the same individual based on the increased bone volume fraction and increased trabecular thickness. The mineral density in these lesions, however, is reduced and may render this area to be mechanically compromised, and thus susceptible to attrition.

## Introduction

Osteoarthritis (OA) is best modeled as a disease of organ failure, in which injury to one joint component leads to damage of other components, and collectively to joint failure and the clinical manifestations of OA. Using magnetic resonance imaging (MRI) as a measurement tool, we have previously demonstrated that bone marrow lesions (BMLs) are an important source of OA symptoms and are also involved in the etiopathogenesis of the disease [1-4]. BMLs are characterized as ill-defined hyperintensities seen on short T1 inversion-recovery images and on fat-suppressed proton density and T2-weighted fast spin echo magnetic resonance images [5].

Findings from the Boston Osteoarthritis of the Knee Study, a natural history study of knee osteoarthritis, have demonstrated that BMLs are strongly associated with the presence of pain in knee osteoarthritis [1], are potent predictors of progression on radiographs [2], and are also predictive of cartilage loss measured semiquantitatively on MRI [4]. There are conflicting data, albeit from smaller studies with different methods, suggesting no relation of BMLs to pain [6,7]; however, the balance of data would support a strong relation of BMLs to pain. Fifty-seven percent of knees in the Boston Osteoarthritis of the Knee Study symptomatic knee OA cohort had a BML at baseline; and of these lesions, 99% remained the same or increased in size at follow-up. Knee compartments with a higher baseline BML score and knee compartments with an increase in BML size were both strongly associated with further worsening of cartilage score. Enlarging or new BMLs occurred mostly in malaligned limbs on the side of the malalignment.

In the clinical research setting, it is clear that MRI of BMLs is useful in that these lesions can be used to identify persons at highest risk for compartment-specific OA progression and those with increased likelihood of having symptoms. Given the strong relationship between BML and mechanical alignment, local mechanical factors may predispose to the development of these lesions.

BMLs in osteoarthritic knees display a number of noncharacteristic histologic abnormalities. In a study by Zanetti and colleagues, 16 consecutive patients referred for total knee replacement (TKR) were examined with sagittal short-inversion-time inversion-recovery and T1-weighted and T2-weighted turbo spin-echo MRI 1 to 4 days before surgery [5]. Tibial plateau abnormalities on magnetic resonance images were compared quantitatively with those on histologic maps. The BMLs (identified as ill-defined and hyperintense on short T1 inversion-recovery images and hypointense on T1-weighted magnetic resonance images) mainly consisted of normal tissue (53% of the area was fatty marrow, 16% was intact trabeculae, and 2% was blood vessels) and a smaller proportion of several abnormalities (bone marrow necrosis (11% of area), necrotic or remodeled trabeculae (8%), bone marrow fibrosis (4%), bone marrow edema (4%), and bone marrow bleeding (2%)). Importantly, edema was not a major constituent of MRI signal intensity abnormalities in these knees.

BMLs have also been found at histologic examinations performed after core decompression in the proximal femora with abnormal MRI findings [8]. MRI–histologic correlation studies of these lesions have demonstrated fat cell destruction and fibrovascular regeneration in the lesion area [9]. In addition, histologic samples obtained in two patients with MRI signal abnormalities in the tibia (that is, similar to those termed here as BML) revealed focal marrow fibrosis and new bone formation, with foci of devitalized bone [10], suggestive of increased remodeling.

These small studies have provided initial insights into the pathology of these lesions although a true understanding of their structure is lacking. The trabecular structure of subchondral bone has been shown previously to be altered in osteoarthritic knees as compared with healthy knees [11]. Specifically, increased bone volume fraction and trabecular thickness, and decreased structure model index (indicating a more plate-like as opposed to rod-like structure), has been observed in the subchondral region of osteoarthritic knees [11]. Whether BMLs themselves are associated with particular abnormalities in trabecular structure, however, is not known at present.

We would hypothesize that BMLs in osteoarthritic knees represent local areas of increased remodeling in subchondral bone, and that contained within the lesion are alterations in trabecular structure. This hypothesis needs to be clarified if we are to maximize our understanding of these lesions, as potentially this knowledge could lead to the definition of therapeutic strategies for the treatment of both the symptoms and structural deterioration associated with knee OA. The aim of the present study was to evaluate the trabecular structure of subchondral bone in regions with and without BMLs using bone histomorphometry.

## Materials and methods

### Study population

We recruited six, postmenopausal, female subjects (age range 48 to 90 years, body mass index range 24.4 to 38.7) with predominantly medial tibiofemoral compartment OA (one participant had predominantly lateral tibiofemoral OA) who were on a waiting list for TKR. The visual analog scale pain in the signal knees of participants ranged from 50 to 100, and the Kellgren and Lawrence grade in that knee ranged from grade 3 to grade 4.

The institutional review board of Boston University Medical Center approved the study. Informed consent was obtained from all study participants.

### Magnetic resonance imaging

Subjects had a MRI scan performed of their study knee prior to TKR (within 2 weeks of their surgery date) on a 1.5 T scanner with dedicated extremity coil (1.5 T Twin Speed Excite scanner; GE Healthcare, Waukesha, WI, USA). The MRI examination consisted of two localizer scans, a sagittal proton density/T2-weighted fat-suppressed series, and a high-resolution coronal three-dimensional SPGR spoiled gradient recalled acquisition with water excitation. The following imaging sequence was used for BML localization on each patient: sagittal dual-echo fast spin echo fat suppressed with TR/TE of ~4,000 milliseconds/15 milliseconds, 60 milliseconds, 2.5 to 3 mm slices, no skip/gap, 256 × 256 matrix, 12 cm field of view (for distal femur and proximal tibia), and acquisition time 4.50 minutes.

Images from the MRI visit of each participant were obtained and uploaded for analysis using Efilm Workstation Software (Merge Healthcare, Milwaukee, WI USA). An axial map of the tibial plateau delineating the precise location of the BML was generated from these images in order to facilitate specimen harvest.

### Specimen harvest

**Figure 1:**
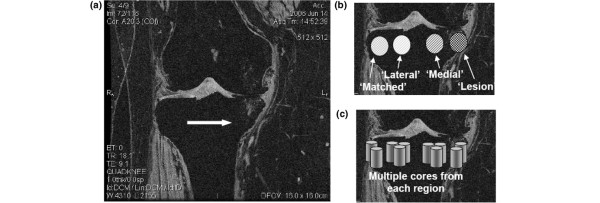
Representative core sampling map as applied to the tibial plateau of a study participant. **(a)** Bone marrow lesions (BML) identified in the medial tibial plateau (arrow). **(b) **Regions from the BML area, from another area within the medial tibiofemoral compartment not affected by BMLs, and from the lateral tibiofemoral compartment as well as from matched locations from the lateral compartment were defined. **(c) **Multiple cores were machined from each region.

At the time of the TKR the tibial plateau was removed as a block with an osteotome. The medial and lateral tibial plateaus were identified and the specimen was transferred immediately to the laboratory for specimen harvest. The BML map on the axial MRI was provided to the laboratory technician, who overlaid this map on the specimen prior to coring. Depending on the size of the BML, between four and 10 cylindrical cores from the medial and lateral tibial condyle were obtained from each specimen using a 6 mm internal diameter trephine (34 cores in total). The locations of the cores within each tibial plateau were specified according to a predefined algorithm (Figure 1) to ensure consistent harvesting between individuals. Cores were taken from the BML area, from another area within the medial tibiofemoral compartment not affected by BML, and from the lateral tibiofemoral compartment as well as from matched locations from the lateral compartment. Core length ranged from 0.3 to 8.2 mm and contained both the cortical-like subchondral plate and some underlying subchondral bone.

### Micro-computed tomography

Specimens were fixed for 5 days in 4% paraformaldehyde in PBS at 4°C. They were then scanned in a high-resolution, desktop microcomputed tomography system (75 kVp, 140 mA, 200 ms integration time; Scanco μCT40; Scanco Medical AG, Basserdorf, Switzerland) at a resolution of 12 microns/voxel. Reconstructed three-dimensional images were segmented using a global threshold determined by an iterative technique [12].

The bone volume fraction and average tissue mineral density, or the degree of mineralization, were calculated for the entire core (subchondral plate and subchondral trabecular bone). To compute the mineral density, the X-ray attenuation of each voxel was converted to mineral density using a calibration curve that was generated from a scan of a set of five hydroxyapatite phantoms of known density (0, 100, 200, 400, and 800 mg hydroxyapatite/cm) provided by the system manufacturer. The tissue mineral density was calculated only for voxels exceeding the threshold (that is, only for voxels occupied by mineralized tissue) – and to minimize partial volume effects, a two-voxel-thick layer was excluded from all trabecular surfaces.

For each core the region containing only trabecular bone was identified manually, and the following structural parameters were quantified: bone volume fraction, trabecular number, trabecular spacing, trabecular number, structural model index (SMI), connectivity density, and degree of anisotropy. The tissue mineral density was also calculated for the trabecular region.

We note that for the analyses of subchondral trabecular bone we required the superior–inferior length of the region containing only trabecular bone to be at least 5 mm. This ensured that the region analyzed would contain a sufficient number of trabeculae for adequate sampling of the trabecular structure. This length criterion resulted in exclusion of 12 cores (one to four cores per donor). Finally, the thickness of the subchondral plate was calculated as the average of measurements made from the microcomputed tomography image data at four equally spaced locations across the superior surface of the plate.

### Statistical analysis

Cores were classified according to location: lesion area (lesion); contralateral (medial or lateral, depending on which compartment contained the lesion) compartment, location matched to lesion area (matched); medial compartment, outside the lesion area or matched area (medial); and lateral compartment, outside the matched area or lesion area (lateral). Repeated-measures analyses of variance with Tukey *post hoc *tests were used to determine differences in bone structure and tissue mineral density among the four locations. When multiple cores were available from a given location for a given donor, each core was treated as an individual measurement for the statistical analyses; measurements were not averaged prior to the analyses of variance.

## Results

We collected specimens from six postmenopausal female OA patients following MRI scan and total knee joint replacement. Histomorphometry measures were obtained on bone core samples from medial (or lateral) tibia affected by BMLs and bone medial (or lateral) regions unaffected by BMLs as well as from control regions from the lateral (or medial) tibial plateau. In order to compare histomorphometric parameters and delineate features specific to BML while controlling for medial-specific and lateral-specific bone features, we categorized bone samples as described above (Figure 1): lesion (bone sample core obtained from medial or lateral tibia affected by BMLs), matched, medial and lateral. The bone volume fraction, trabecular thickness, trabecular spacing, tissue mineral density and other architectural features of BML-affected bone tissue cores were therefore compared with control regions contralateral to the BML and with unaffected regions immediately adjacent to the BML.

**Figure 2:**
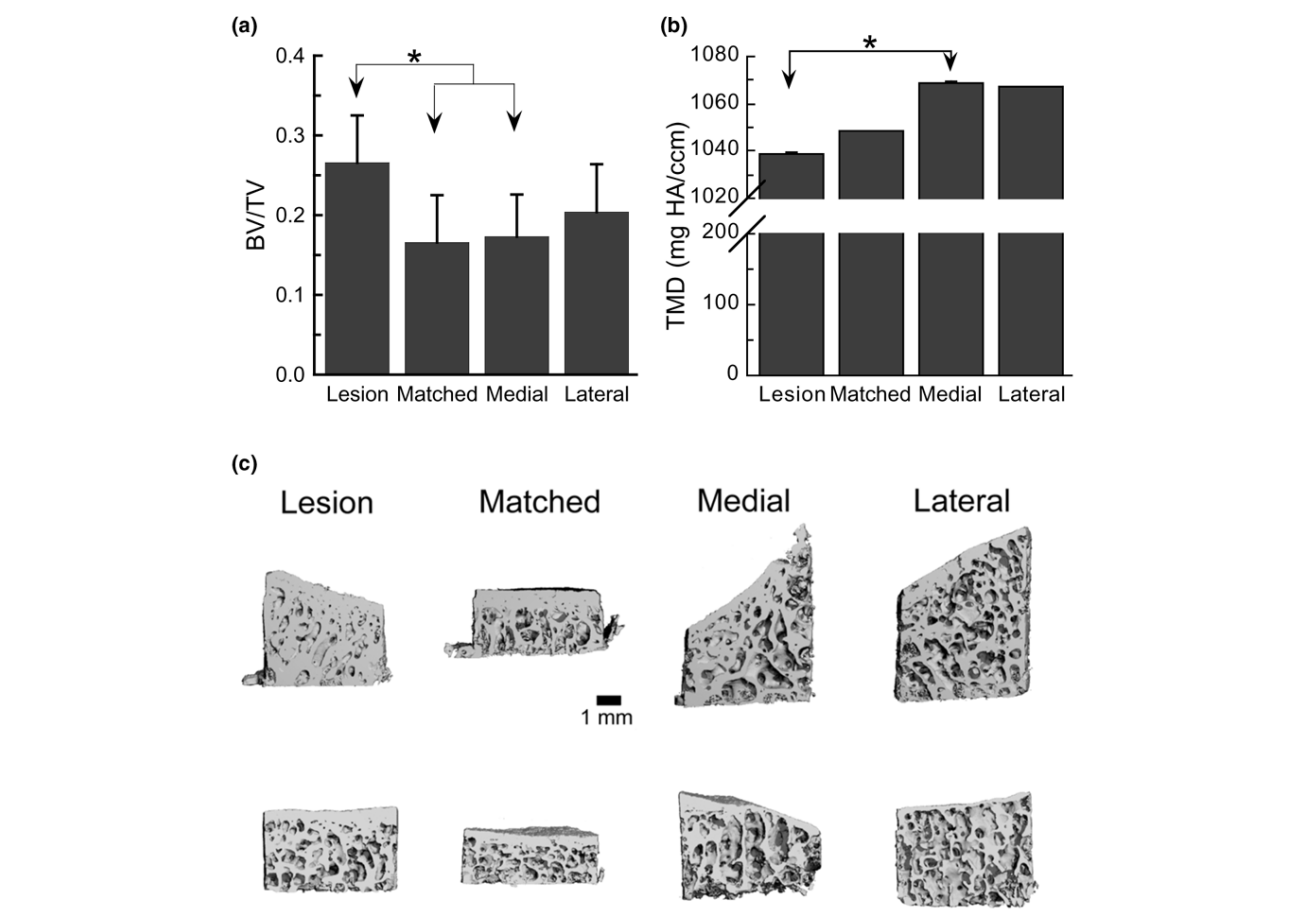
Bone volume fraction and average tissue mineral density for four locations from the entire core. **(a) **Bone volume fraction (BV/TV) and **(b) **average tissue mineral density (TMD) for the entire core for each of the four locations. HA, hydroxyapatite. Each bar represents the mean, and error bars represent one standard deviation. *Significant differences between groups (*P *< 0.05). Cores from the lesion area exhibited the highest volume fraction but lowest mineral density. **(c) **Longitudinal cut-away views of cores from each of the four locations. Each row contains cores from one donor.

**Figure 3:**
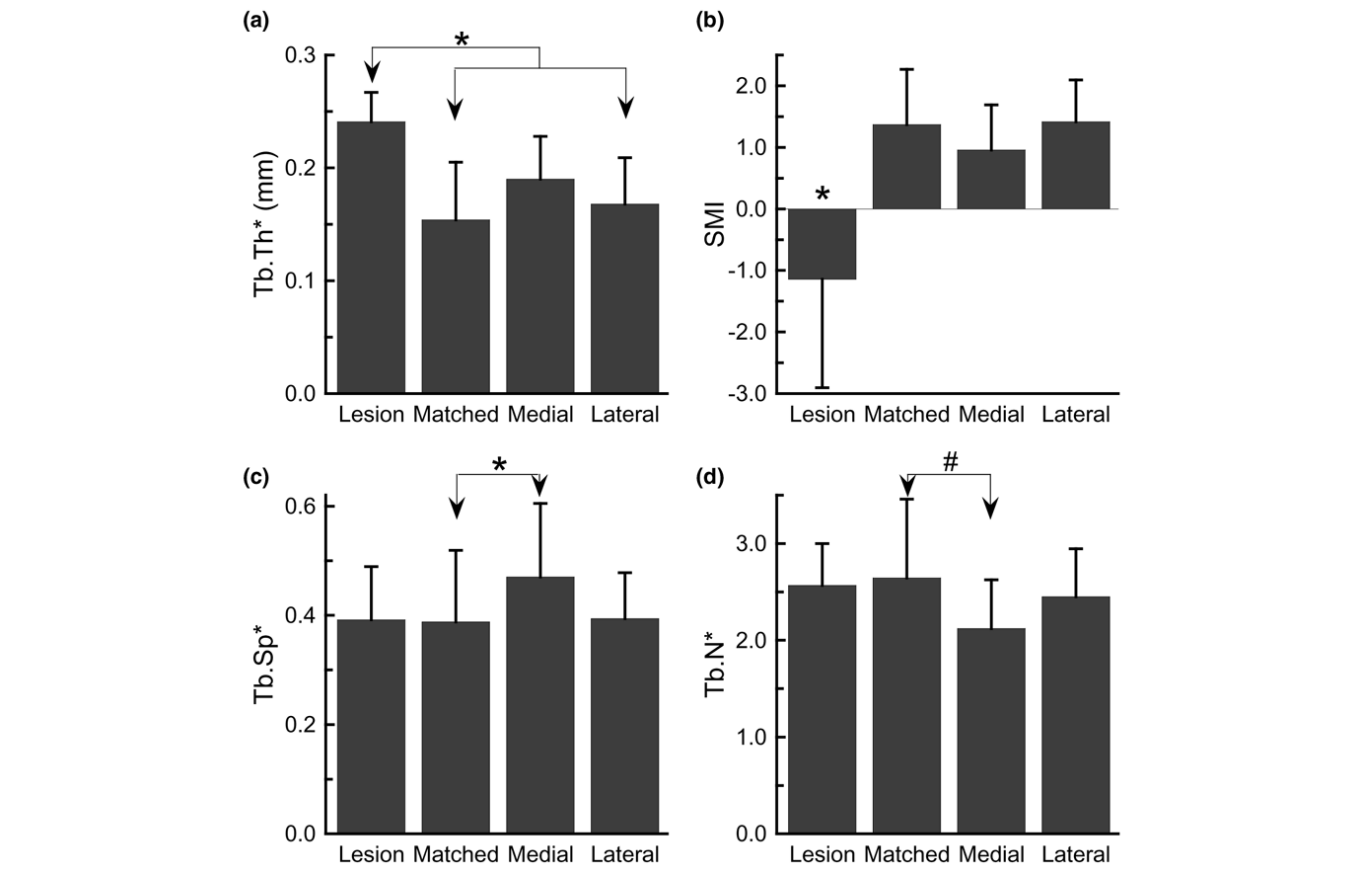
Quantitative measures of the trabecular structure for each of the four locations. **(a) **Trabecular thickness (Tb.Th*). **(b) **Structure model index (SMI). **(c) **Trabecular spacing (Tb.Sp*). **(d) **Trabecular number (Tb.N*). Cores from the lesion area exhibited the highest Tb.Th* but lowest SMI. Differences in trabecular structure were also noted between the matched and medial locations. Each bar represents the mean, and error bars represent one standard deviation. *Significant differences between groups (*P *< 0.05). ^#^A trend (0.05 ≤ *P *< 0.10).

Cores from the lesion area displayed increased bone volume fraction but reduced tissue mineral density (*P *< 0.04; Figure 2). With respect to the subchondral trabecular structure, the samples from the lesion area exhibited increased trabecular thickness as compared with samples from the matched area and lateral location (*P *= 0.02; Figure 3a). The subchondral bone in the lesion area was also markedly more plate-like than the bone in the other three locations, as evidenced by the lower value of the SMI (*P *= 0.009) (Figure 3b). Other differences in structure that were noted were increased trabecular spacing (*P *= 0.02) and a trend (*P *= 0.07) towards decreased trabecular number in the cores from the medial location as compared with matched location (Figure 3c,d). No differences among locations were found in connectivity density, degree of anisotropy, or tissue mineral density of the subchondral trabecular bone (*P *> 0.10). In addition, no differences among locations were found in the subchondral plate thickness (*P *= 0.10) (Table 1).

**Table 1:** Subchondral plate thickness by location

Location	*n*	Mean (standard deviation) (mm)
Lateral	11	0.49 (0.14)
Lesion	5	0.77 (0.24)
Matched	5	0.56 (0.10)
Medial	9	0.66 (0.34)

## Discussion

Research into the etiology and progression of knee OA has focused on the destruction of articular cartilage. However, it is clear that knee OA is an organ-level failure of the joint and involves pathological changes in subchondral bone as well as in articular cartilage [13]. We have found that BMLs, which are strongly associated with OA symptoms and disease progression, have specific changes in bone mineralization and trabecular structure. The BML area, when compared with bone samples within the same knee but outside the lesion area, appeared to be sclerotic, based on the increased bone volume fraction and increased trabecular thickness. The trabecular architecture within the lesions was also more plate-like; however, the tissue mineral density was reduced relative to medial tibial bone outside the BML.

Our findings are consistent with those of prior work suggesting that hypomineralization of trabecular bone in OA occurs subjacent to the thickened cortical plate [14-16]. This reduced mineralization is possibly linked to abnormal bone cell behavior in OA joints, reported as imbalances in bone resorption, bone formation or both [17]. Recent studies have confirmed that increased bone resorption plays an integral role in the disease process, with increased levels of bone resorption markers, including type I collagen [18] and deoxypryidinoline [19], reported in patients with radiographic evidence of knee OA. Urinary excretion of pyridinium cross-links is significantly increased in patients with large joint OA and hand OA, suggesting an increased rate of bone turnover [20]. Data from the population based Chingford study demonstrated that urinary collagen cross-link excretion (urine C-telopeptide and N-telopeptide) levels were significantly elevated in knee OA subjects [21]. Elevated levels of urinary N-telopeptide indicate elevated human bone resorption [22], and our own data suggest their levels are increased in persons with BMLs [23]. It is important to note that these findings are not consistent with previous research such as that showing the bone turnover markers were decreased in patients with knee OA compared with control individuals (-36%, -38%, and -52%, *P *< 0.0001 for serum osteocalcin, serum and urinary C-terminal telopeptide of type 1 collagen, respectively) [24].

Moreover, our data are in agreement with previous findings from early OA tibial bone specimens. This previous research indicates that the trabeculae in the medial compartment of OA joints are significantly thicker and more plate-like than normal trabeculae [11,25,26], but that the affected trabecular bone is less stiff than normal bone at both the apparent level [25] and the tissue level [27].

Our data also extend previous findings, however, in that they uniquely identify OA BMLs as foci of bone architecture pathology. While prior studies have compared the subchondral trabecular structure between osteoarthritic knees and normal knees [11,25,27], the present study provides a comparison of the architecture in BML-affected area with that in other areas within the same tibial plateau. Results of the latter comparison indicate that the affected region is one of pronounced abnormalities in structure and mineralization. These structural abnormalities are evident in the quantitative analysis of trabecular architecture (Figure 3) as well as through qualitative examination of the three-dimensional images of the specimens (Figure 2c). In particular, SMI values in the lesion areas ranged from -2.20 to 0.89 (mean = -1.14), while those in the other three areas ranged from -0.38 to 2.78 (mean = 1.24). A SMI of 3 indicates an ideal cylindrical rod structure, an SMI of 0 indicates an ideal plate structure, and values less than zero indicate a structure in which the plates are curved and begin to close off the pores from one another [28]. The results of the present study therefore indicate that the BML architecture is an extreme representation of the changes that occur throughout the affected compartment in OA.

The changes in trabecular structure within the BML-affected area are in opposition to the typical age-related changes in trabecular structure in the proximal tibia that involve trabecular thinning and progression from a plate-like structure to a rod-like structure [29-31]. These OA-related changes, however, do not necessarily imply any mitigation of age-related degradation in mechanical properties.

Previous studies have found that while bone volume fraction and bone mineral density can increase substantially in the early stages of OA [27,32,33], these changes are associated with either no change or a slight decrease in apparent Young's modulus and compressive strength [25,27]. Studies on the microstructural and mechanical properties of tibial cancellous bone by Ding and colleagues have revealed that the SMI and the bone volume fraction can be primary determinants of cancellous bone mechanical properties [34]. Importantly, plate-like cancellous bone is associated with increased relative strength relative to rod-like bone; however, the converse is true in osteoarthritic bone [11]. In addition, studies on cancellous bone from the femoral head of OA and osteoporosis patients revealed that the stiffness of osteoarthritic bone increased more slowly with apparent density and that its material density was significantly reduced (associated with 12% reduction in mineral mass fraction). Intriguingly, the authors reported there was also greater site-to-site variation of both apparent and material density in the osteoarthritic bone, suggesting an altered sensitivity to applied load [35].

These collective findings have led to the hypothesis that the trabecular tissue is mechanically compromised in OA, probably as a result of poor trabecular organization [11,27]. The reduced tissue mineral density and mineral:collagen ratio in OA tissue [15,36] is consistent with this hypothesis. Although the cellular basis for the reduced mineralization is not clear at present, prior studies have noted in OA that increases in osteoid volume occur as a result of trabecular thickening that is usually not accompanied by increased bone mineralization [16,37].

**Figure 4:**
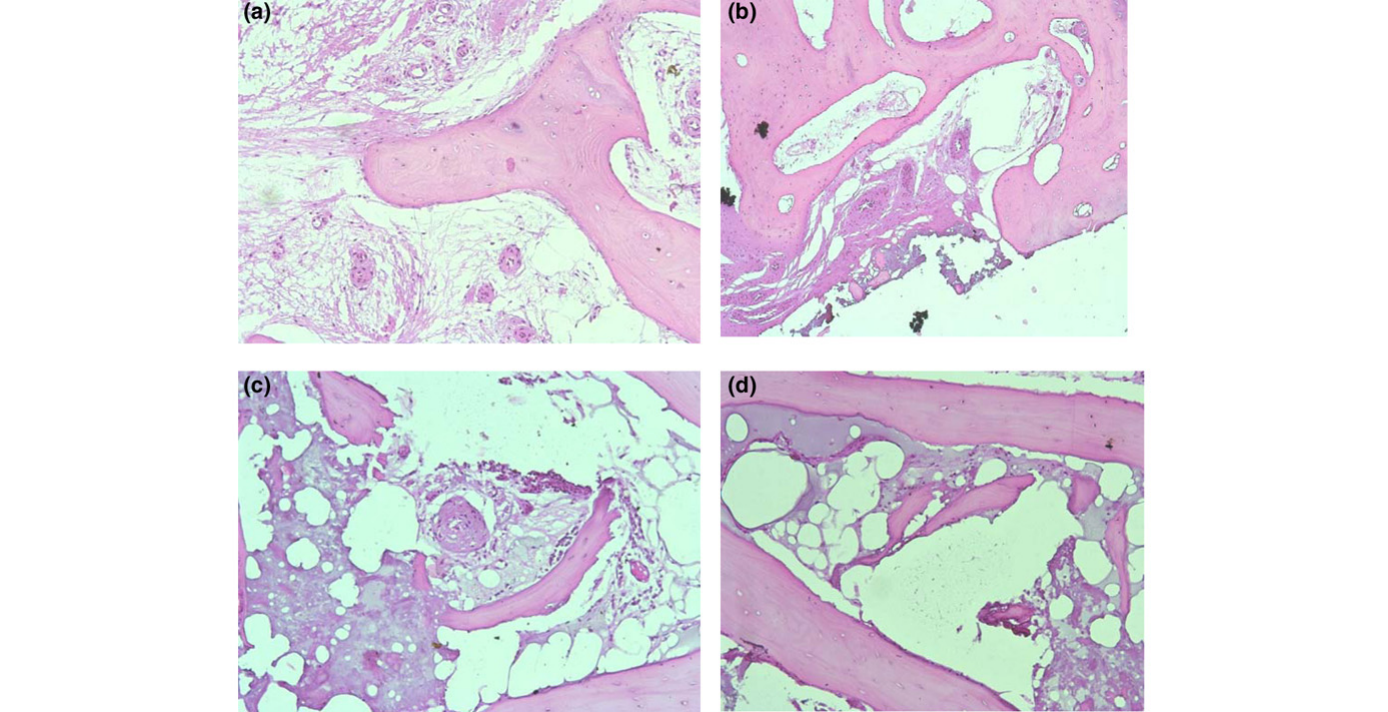
Histopathological analyses of bone marrow lesion cores indicating a mixed pathology. **(a) **Diffuse granulation reaction in the marrow compartment. All blood vessels show signs of secondary remodeling with thickened walls. Some vessels show evidence of focal fibrinoid adhesion to the endothelium. **(b) **High-power view of focal granulation reaction. **(c) **Regional granulation reaction continuous with a focal fibrinoid reaction with thrombus inclusions. There is evidence of a low-grade inflammation peripheral to the fibrinoid edge. The marked vessel remodeling and the presence of fibrinoid inclusions in the granulation zone are consistent with a focal infarction. **(d) **Vascular leak with multiple thrombus inclusions. There is fibrinoid occupation and casting of the marrow stroma.

The histological analyses for this study are ongoing. Preliminary data from parallel histopathological studies on OA BML cores indicate a mixed pathology within the BML including granulation, edema, diffuse necrosis, fibrinoid deposition and hyperplasia of blood vessel walls (see Figure 4). Some of these features have previously been reported [5,9]; however, taken as a whole, our preliminary data point towards pathology consistent with a localized infarction reaction. Although these data need further validation and cross-reference versus additional tissue sets, our early findings may point to towards a localized oxygen deficit in the BML – which may contribute to the focal bone remodeling reactions observed in OA BMLs.

There are a number of important limitations of the present study that warrant mention. This is a small sample of six postmenopausal women and thus the findings cannot be generalized to men and those with OA in other parts of the joint. Further, given the small sample size, this work should be extended and replicated in other samples. An additional rate-limiting step in this approach is that the depth of the cut in the tibial plateau from TKR provides small specimens that did not always permit quantification of the subchondral trabecular structure. Despite these limitations, however, our data are striking in that statistically significant differences in several histomorphometric parameters were identified.

## Conclusion

We have localized specific changes in bone mineralization, remodeling and defects within BML features that are adjacent to the subchondral plate. The mineral density of these BMLs is reduced, and they appear to be sclerotic compared with unaffected regions from the same individual based on the increased bone volume fraction, increased trabecular thickness, and decreased SMI. Further work is required to determine how these changes in composition and structure affect the mechanical properties of the BML subchondral bone, and thus whether these changes render the bone susceptible to attrition. In addition, future studies are required to evaluate whether these observations are caused by an increase in synthesis or a decrease in resorption of the bone, and how these relate to histopathological features.

## Abbreviations

BML: bone marrow lesion; MRI: magnetic resonance imaging; OA: osteoarthritis; PBS: phosphate-buffered saline; SMI: structure model index; TKR: total knee replacement.

## Competing interests

The authors declare that they have no competing interests.

## Authors' contributions

DJH conceived of the study, participated in acquisition of the data, data analysis and interpretation, and manuscript preparation, and approved the final manuscript. LG, TAE, and RAM participated in data analysis and interpretation, manuscript preparation and approved the final manuscript. GB, ADD, ZDM, PN, EFM participated in acquisition of the data, data analysis and interpretation, and manuscript preparation, and approved the final manuscript. MF and SJ participated in acquisition of the data and manuscript preparation.
